# An evolutionary explanation for the presence of cancer nonstem cells in neoplasms

**DOI:** 10.1111/eva.12030

**Published:** 2012-11-26

**Authors:** Kathleen Sprouffske, C Athena Aktipis, Jerald P Radich, Martin Carroll, Aurora M Nedelcu, Carlo C Maley

**Affiliations:** 1 Genomics and Computational Biology Program, Department of Biology, University of PennsylvaniaPhiladelphia, PA, USA; 2 Center for Evolution and Cancer, Helen Diller Family Comprehensive Cancer Center, Department of Surgery, University of California San FranciscoSan Francisco, CA, USA; 3 Department of Ecology and Evolutionary Biology, University of ArizonaTucson, AZ, USA; 4 Department of Psychology, Arizona State UniversityTempe, AZ, USA; 5 Clinical Research Division, Fred Hutchinson Cancer Research CenterSeattle, WA, USA; 6 Division of Hematology and Oncology, University of PennsylvaniaPhiladelphia, PA, USA; 7 Department of Biology, University of New BrunswickFredericton, NB, Canada

## Abstract

Contrary to conventional views that assume all cells in a neoplasm can propagate the tumor, the cancer stem cell hypothesis posits that only a fraction of the cells (the cancer stem cells) can act as tumor-propagating cells, while most of the tumor is composed of cells with limited replication potential. Here, we offer an evolutionary approach to this controversy. We used several evolutionary, computational models to investigate cancer cell dynamics and conditions consistent with the stem cell hypothesis. Our models predict that if selection acts at the cell level, neoplasms should be primarily comprised of cancer stem cells, in contrast to experimental data indicating that neoplasms contain large fractions of cancer nonstem cells. We explore several solutions explaining the paradoxical existence of cancer nonstem cells in neoplasms, including the possibility that selection acts at the level of multicellular proliferative units.

## Introduction

Cancer is a by-product of multicellularity. During the transition to multicellularity, single-celled organisms cooperated to form stable units favored by natural selection (Maynard Smith and Szathmáry 1997). Further fitness advantages were gained when cells in these cooperative units specialized into reproductive and nonreproductive roles (e.g., germ and somatic cells), resulting in multicellular groups in which individual cells contributed to the fitness of the newly emerged multicellular individual at the expense of their own fitness (Buss 1987; Maynard Smith and Szathmáry 1997). In this context, cancer occurs when some cells acquire mutations that stop promoting the fitness of the multicellular organism by increasing their own cell division or survival relative to normal cells (e.g., self-sufficiency in growth signals, evading apoptosis, limitless replicative potential) (Nowell 1976; Hanahan and Weinberg 2000, 2011; Merlo et al. 2006; Greaves and Maley 2012). At short timescales, these ‘selfish’ cells can be selected for (known as somatic evolution) and form neoplasms than can eventually kill the multicellular individual. Consequently, several mechanisms that suppress somatic evolution in adult tissues have evolved (Nowell 1976; Pepper et al. 2007).

One mechanism that suppresses somatic evolution, and thus neoplasm formation, is the division of tissues into proliferative units with a few somatic stem cells in each unit (Cairns 1975). Somatic stem cells can either divide symmetrically to replenish their numbers or differentiate asymmetrically to generate the transient cells and terminally differentiated cells that make up the bulk of the tissue (Pepper et al. 2007). Transient cells can divide multiple times, becoming progressively more differentiated. Somatic evolution is suppressed because only mutations that occur in stem cells are retained, while most other mutations are lost. Observations of markers of somatic differentiation hierarchy in neoplasms and experimental verification of functional differences between neoplastic cells sorted by these markers of differentiation (Bonnet and Dick 1997) led to the hypothesis—known as the cancer stem cell hypothesis—that tumors are organized in a manner similar to that of normal tissue. Lineage-tracking experiments in mouse glioblastoma, intestinal tumors, and squamous skin tumors (Chen et al. 2012; Driessens et al. 2012; Schepers et al. 2012) have demonstrated that cancer stem cells give rise to differentiated, nonstem cancer cells.

**Figure 1:**
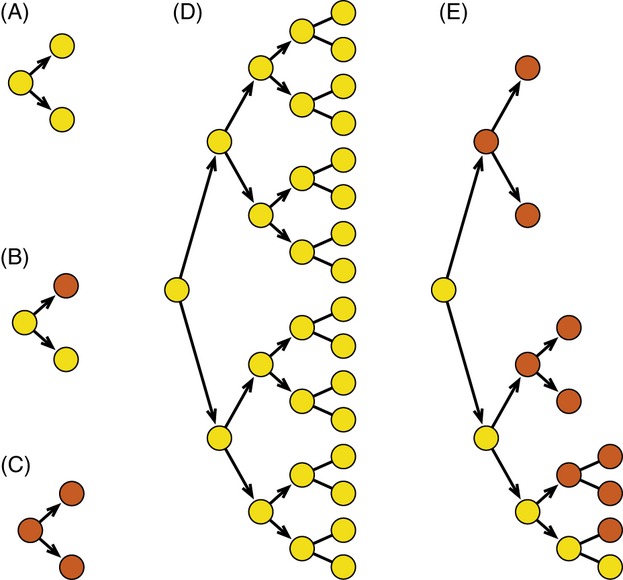
Models of cancer cell division and differentiation. Cancer stem cells, or tumor-propagating cells, are light yellow; transient cells are dark orange. The color version is available online. A tumor-propagating cell can divide (A) symmetrically, in which both daughters remain tumor-propagating cells, or (B) asymmetrically, in which one daughter remains a tumor-propagating cell and the other differentiates. (C) When a transient cell divides, both daughter cells become more differentiated. The relative proportion of a tumor-propagating clone's symmetric-to-asymmetric divisions affects the number of tumor-propagating cells in a neoplasm. (D) Clones of tumor-propagating cells that only divide symmetrically increase the tumor-propagating population size exponentially and (E) those that only divide asymmetrically do not. Because tumor-propagating cells may undergo apoptosis, they must divide symmetrically in some cases to maintain homeostasis.

Like somatic stem cells, cancer stem cells are thought to divide symmetrically to produce two cancer stem cells, or to divide asymmetrically to produce one cancer stem cell and one transient cell (Maenhaut et al. 2010) (Fig. 1). The ability of glioma stem cells to self-renew by symmetric division and produce differentiated cells by asymmetric division has been shown in single-celled experiments (Lathia et al. 2011). Using an *Erb2* transgenic model of breast cancer, increasing frequency of symmetric and self-renewing cancer stem cell divisions led to high proportions of cancer stem cells (Cicalese et al. 2009). Alterations in several genes, including mutations in a number of tumor suppressors, have been shown to affect whether cells can undergo polar cell division (Royer and Lu 2011).

Cancer stem cells are experimentally defined as a fraction of reproducibly isolated malignant cells that can survive xenotransplantation and, once engrafted, can generate a new neoplasm that recreates the full heterogeneity of cell surface markers or phenotypes found in the original neoplasm (Maenhaut et al. 2010). Further, the fraction should be capable of surviving serial passaging, which demonstrates the capability to self-renew. The debate surrounding cancer stem cells originates from the widely varying observations of cancer stem cell numbers as well as their functional roles within the neoplasm. The proportion of a neoplasm thought to be cancer stem cells differs by the type of cancer and experimental methods (Rosen and Jordan 2009; Visvader and Lindeman 2012) and even between neoplasms [e.g.,(Sarry et al. 2011)]. For instance, in melanoma, a stem cell frequency of 25% was reported (Quintana et al. 2008), while in acute myeloid leukemia (AML) or colon cancer, only 1 in 10^4^ or 10^5^ cells have been identified as cancer stem cells (Lapidot et al. 1994; O'Brien et al. 2007). Because there is controversy over whether the structure of differentiation in a neoplasm replicates differentiation in normal tissues, we prefer the term ‘tumor-propagating cell’ to emphasize the functional role of cancer stem cells in propagating neoplasms (Maenhaut et al. 2010).

Here, we use an evolutionary framework and several models to investigate the conditions and the fundamental assumptions underlying the cancer stem cell hypothesis—that there is a fraction of cells, the tumor-propagating cells that can self-renew by symmetric division, while all other cells eventually terminally differentiate. To do so, we constructed individual-based models of cell evolution within a neoplasm and a stage-structured analytical model of cell growth and differentiation. According to the cancer stem cell hypothesis, we posit that there are tumor-propagating and transient cells and that the probability that any given tumor-propagating cell will divide symmetrically (and produce two tumor-propagating cells) rather than asymmetrically is a heritable trait that can evolve. Briefly, an individual-based model is a stochastic computational simulation technique in which we explicitly encode cell behaviors (cell division, mutation, differentiation, and death) and traits (probability of symmetric division) in a computational model, simulate populations of cells, and record the resulting system dynamics. We allowed the trait of the probability that a tumor-propagating cell could divide symmetrically to evolve by the mutation at cell division. Thus, each cell in a simulation can have a different probability for symmetric division, and those cells with the most favorable trait value are expected to dominate the population over time. Using this simulation technique, we encoded the basic formulation of the cancer stem cell hypothesis and observed the proportion of tumor-propagating cells that resulted.

## Materials and Methods

### Individual-based model

The full methods are presented in standard format for individual-based models (Grimm et al. 2006) in the Supplementary Methods. Both tumor-propagating cells and transient cells were modeled. Experimentally, cell surface markers are used to identify subpopulations of tumor-propagating cells [e.g., (Lapidot et al. 1994; Bonnet and Dick 1997; O'Brien et al. 2007; Quintana et al. 2008; Boiko et al. 2010; Roesch et al. 2010; Taussig et al. 2010), reviewed in (Visvader and Lindeman 2012)]. The precise nature of our model allows us to know exactly which are the tumor-propagating cells and the transient cells. In this way, we have removed all complications stemming from uncertainty associated with identifying tumor-propagating cells within our model. Each day in the simulation, a cell had the opportunity to die, divide, or do nothing. Transient cells could divide symmetrically a parameterized number of times before undergoing apoptosis or senescence. Except where noted, transient cells could not dedifferentiate into tumor-propagating cells. The probability that a tumor-propagating cell divided symmetrically or asymmetrically was determined by a mutable cell intrinsic trait that could evolve. Thus, each cell division in simulations of the model produced either two tumor-propagating cells in a symmetric division or one tumor-propagating cell and one transient cell in an asymmetric division. We recorded the number of cells that divided symmetrically and asymmetrically within a generation and used these data in our analyses.

### Overview of Simulations

Unless otherwise specified, all simulations were run using the default parameters specified in Table S2. For each combination of parameters, 50 simulations lasting 10 years were run. The parameter values in some cases were not suitable to sustain a cell population, and so the actual number of simulations that survived 10 years is reported. Control simulations were run in which no new mutation in the symmetric division trait was allowed to occur, while otherwise using the default parameters. Differences in the mean probability for symmetric division between the control simulations and across all other parameter values were quantified using a *t*-test with a Bonferroni adjusted significance threshold (Ewens and Grant 2005).

## Results

### Tumor-propagating cells are favored by natural selection if selection acts at the cell level

**Figure 2:**
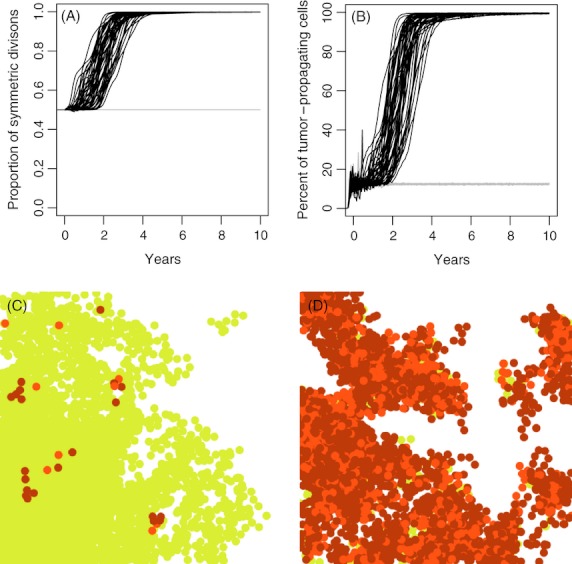
The effects of natural selection on tumor-propagating cells' proportion of symmetric divisions and the resulting tumor composition. (A) For a representative evolving population of neoplastic cells (black lines), natural selection increases the population average of tumor-propagating cells' proportions of symmetric divisions over time from 0.5 to 0.99894 (mean, *n* = 49, SEM = 0.00003). When a population cannot evolve (gray lines), the population average of tumor-propagating cells' proportions of symmetric divisions does not change. (B) The percent of tumor-propagating cells in a bulk tumor increases over time for these evolving populations (black lines) to 99.89% (mean, *n* = 49, SEM = 2.60%), while it remains constant for populations that cannot evolve (gray lines) at 12.42% (mean, *n* = 49, SEM = 1.28%). The mutation rate is the only parameter that varies between these two types of simulations (μ = 1 × 10^−3^ for black lines, μ = 0 for gray lines); otherwise, default parameter values were used (see Table S3). Fifty simulations were conducted per condition, although 1 simulated neoplasm per condition died before 10 years. (C) After 10 years of simulated evolution of tumor-propagating cells' probability of symmetric division, the neoplasm was primarily comprised of tumor-propagating cells. (D) When symmetric division does not evolve, the neoplasm is comprised primarily of transient cells. Tumor-propagating cells are light yellow, transient cells are dark orange, and the maximum population size = 5000 cells for visual clarity. The color version is available online.

We focused the parameters of our model on a population of AML cancer cells containing both tumor-propagating cells that could have unlimited numbers of divisions and transient cells that had a limited number of divisions before they terminally differentiated. We allowed the probability of symmetric division to mutate at cell division and found that both the proportion of tumor-propagating cell symmetric divisions and the percent of tumor-propagating cells in the neoplasm increase over time due to somatic evolution (Fig. 2A,B). In the end, most tumor-propagating cell divisions were symmetric, and few transient cells remained (Fig. 2C,D). The mean probability of symmetric division increased from the initial value of 0.5 to a final value of 0.99894 (mean, n = 49, SEM = 0.00003), and the mean percent of tumor-propagating cells evolved to 99.60% (mean, n = 49, SEM = 0.01%). While individual cells may evolve a probability of symmetric division value of 1, they are not expected to remain there. New mutations arise that introduce smaller values. The proportion of tumor-propagating cells did not increase under control experiments in which the probability for symmetric division could not evolve (Fig. 2A,B). As expected, increasing the probability for tumor-propagating cells' symmetric division was sufficient to increase the percent of tumor-propagating cells in the neoplasm (Fig. S1A; *R* = 0.97). These results suggest that tumor-propagating cells should expand over time to represent the great majority of the malignant cells.

We obtained similar results when we repeated these experiments across a variety of parameter values, confirming the robustness of our model to the number of transient cell divisions before terminal differentiation, mutation rate, symmetric division trait variability, and maximum neoplasm size (see Supplementary Text, Figs. S1–S3, Table S1). There was a significant difference between the evolved symmetric division levels for all parameter values, except low mutation rates, to the control case of no evolution (*t*-test, *P* < 10^−21^ for all significant values, see Table S1 for parameter values tested and their exact *P* values). While the precise time it takes for tumor-propagating cells to dominate the tumor varies due to model parameters, it takes less time under high mutation rates, high symmetric division rate variability, large neoplasm size, and fewer transient cell stages (Table S2). The general case of time to fixation of mutant alleles has been studied elsewhere (Durrett and Schmidt 2008; Dingli et al. 2007b Beerenwinkel et al. 2007). These studies confirm the robustness of our findings in the biologically reasonable parameter space between neoplasms.

To confirm that tumor-propagating cells with higher symmetric division rates are favored over tumor-propagating cells with lower symmetric division rates for any size of tumor, we used an analytical stage-structured population model (Lefkovitch 1965; Caswell 2001) that allowed exponential growth of the neoplasms. We split the tumor-propagating cells into two separate populations with different symmetric division rates and calculated the asymptotic stage distribution (see Supplementary Methods). We found that the tumor-propagating cell population with lower symmetric division rates went extinct, which confirms the results of our individual-based model. These results are consistent with previous research (Dingli et al. 2007a) that has shown using exact, stochastic simulations that mutant clones with higher rates of symmetric self-renewing cell divisions can expand and take over the tumor.

**Figure 3:**
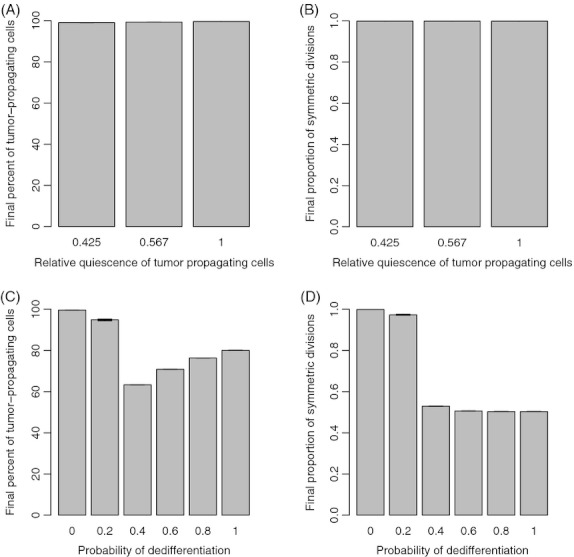
The effects of natural selection on tumor-propagating cells' proportion of symmetric divisions and the resulting tumor composition when tumor-propagating cells are quiescent and dedifferentiation can occur. The relative quiescence of tumor-propagating cells to transient cells resulted in the evolution of (A) high tumor-propagating cell levels and (B) high tumor-propagating cell symmetric division probabilities [*n* = (37,45,50) for relative quiescence = (0.425,0.567,1)]. (C) Under the possibility that transient cells can dedifferentiate at cell division, high levels of tumor-propagating cells are still found, although (D) tumor-propagating cells' symmetric division rates are unchanged if transient cells dedifferentiation is common (*n* = 48 when dedifferentiation is 0; *n* = 50 otherwise). Error bars show standard error of the mean, and 50 simulations were initialized per condition.

Next, we tested to see whether introducing quiescent stem cells, or tumor-propagating cells that divide intermittently, affected the evolution of high levels of symmetric division in tumor-propagating cells. We varied the relative quiescence of tumor-propagating cells to transient cells by varying the probabilities of cell division for transient and tumor-propagating cells. A relatively quiescent value of 0.5 for a simulation means that the probability of tumor-propagating cell division is half that of transient cells. We still observed the evolution of high tumor-propagating cell symmetric division levels and high proportions of symmetric division (Fig. 3). There was a significant difference between the evolved symmetric division levels for a range of relative quiescent values to the control case of no evolution (*t*-test, *P* < 10^−126^ for all values). Thus, natural selection acting at the cell level continues to favor high tumor-propagating cell symmetric division levels, even when tumor-propagating cells are relatively quiescent.

It has been suggested that nonstem cells in cancer may dedifferentiate into cancer stem cells (Gupta et al. 2009), which is consistent with recent observations (Roesch et al. 2010; Taussig et al. 2010). Thus, we addressed whether dedifferentiation changes the outcome of our model by allowing stochastic dedifferentiation of transient cells to tumor-propagating cells at cell division. Our model showed that tumor-propagating cells still dominated the neoplasm, although the mechanism differed at low and high levels of dedifferentiation. For infrequent dedifferentiation, high levels of symmetric division evolved, as before. However, for frequent dedifferentiation, the probability of symmetric divisions evolved significantly, but by very small amounts. Here, the numbers of tumor-propagating cells increased by transient dedifferentiation rather than by the evolution of the symmetric division rate (Fig. 3). For both cases, there was a significant difference between the evolved symmetric division levels and those of the control case of no evolution (*t*-test, *P* < 10^−4^ for all parameter values). Thus, even at higher dedifferentiation levels, most cells are effectively cancer stem cells (including transient cells, which are likely to become tumor-propagating cells).

### Conditions under which cancer nonstem cells may exist

We showed that, within a neoplasm, natural selection acting at the cell level favors the evolution of mechanisms that increase the percent of tumor-propagating cells by selecting for high levels of symmetric division, or through dedifferentiation. Our model predicts that 99.60% of cells in neoplasms should be tumor-propagating cells, but experiments on human tumors observed between 0.001% and 25% cancer stem cells (Lapidot et al. 1994; O'Brien et al. 2007; Quintana et al. 2008). A more recent melanoma study found that up to 41% of cells belong to the tumor-initiating fraction (Boiko et al. 2010).

We address the mismatch between experimental observations and the predictions of our evolutionary model on tumor composition. To do so, we consider four nonexclusive, potential explanations: (i) current assays may underestimate tumor-propagating cell numbers, or the cancer stem cell hypothesis is wrong, (ii) somatic evolution may be occurring too slowly for tumor-propagating cells to take over the tumor, (iii) the propensity for symmetric divisions may not be heritable and is instead conferred by the tumor microenvironment, or (iv) transient cells may boost the fitness of the tumor-propagating cell lineage that produced them or their proliferative units.

First, perhaps, the cancer stem cell hypothesis is wrong. In this case, the assumptions of our model have been violated; namely there is no differentiation hierarchy within a tumor, and all cells in a tumor are tumor-propagating cells. If the cancer stem cell hypothesis is wrong, then current assays significantly underestimate the tumor-propagating cell numbers. Most assays implicitly measure both self-renewal and ability to engraft as a xenotransplant, and so exclude tumor-propagating cells incapable of engraftment under these conditions (Kelly et al. 2007). Improved, more permissive protocols should reveal a higher proportion of tumor-propagating cells as has been seen in AML (Taussig et al. 2008). Except possibly in melanoma, the most permissive systems are still far from identifying every cell in within a neoplasm as capable of propagating a tumor. In breast cancer, Polyak and colleagues have shown the presence of genetic lesions and clonal expansions in the putative transient cells, suggesting extensive self-replication among cells previously thought to be transient cells (Shipitsin et al. 2007; Park et al. 2010). Comparisons between the (epi)genetic clonal structure of tumor-propagating cells and transient cells (Park et al. 2010) could reveal additional deficiencies in the cell surface marker characterization of tumor-propagating cells and lend support for this solution.

Second, transient cells might exist in neoplasms because somatic evolution is not occurring quickly enough for the symmetric division rate to evolve and allow tumor-propagating cells to dominate the neoplasm. We tested this with our model, and as expected, we found that the probability for symmetric division could not evolve under low mutation rates and trait variability (Fig. S2). Under conditions of low variability, we would expect the probability for symmetric division to undergo selection for a longer period of time before reaching the mutation–selection equilibrium. Thus, we would expect to find steadily increasing proportions of tumor-propagating cells in serial engraftment studies until the mutation–selection equilibrium is attained. The experimental evidence is inconclusive. Some experiments in melanoma did not find increasing numbers of tumor-propagating cells (Roesch et al. 2010), while others observed increased engraftment of cells from xenopassaged samples as compared to cells from surgical samples (Boiko et al. 2010). Recent lineage-tracking experiments in mice suggest that the proportion of tumor-propagating cells increases from the precursor benign papilloma to malignant invasive squamous cell carcinoma (Driessens et al. 2012). Because the epigenetic mutation rate is orders of magnitude faster than the genetic mutation rate, cancer is characterized by genomic instability (Hanahan and Weinberg 2000), and other phenotypes like therapeutic resistance readily evolve, we find it unlikely that somatic evolution is too slow for the symmetric division rate to evolve for most tumor types.

**Figure 4:**
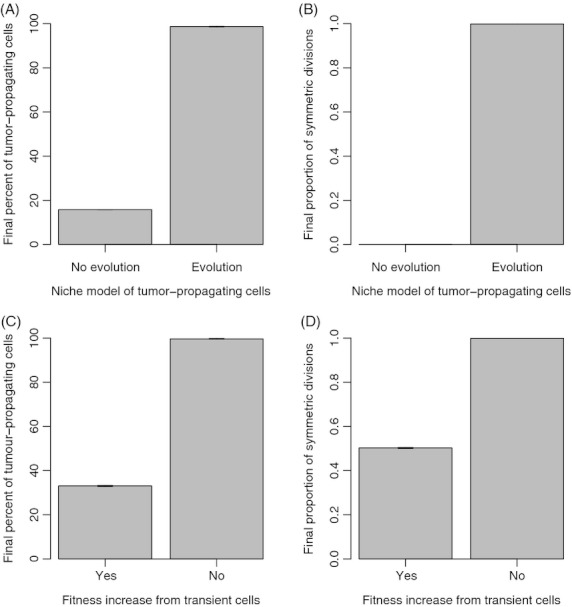
Microenvironment and multi-level selection model extensions. Allowing the microenvironment to determine tumor-propagating cells still resulted in the evolution of (A) high tumor-propagating cell levels and (B) high tumor-propagating self-renewing cell division probabilities. As a control for the microenvironment model, we ran simulations in which the probability for tumor-propagating cell self-renewal could not evolve. (C) The final percent of tumor-propagating cells is smaller when transient cells contribute to the fitness of clonally related tumor-propagating cells than when they do not, and (D) the final proportion of symmetric divisions does not increase (initial value: 0.05, final value: mean = 0.502, SEM = 0.002). Error bars show standard error of the mean, and 50 simulations were initialized per condition (*n* = 50 for all conditions).

Third, the propensity for symmetric division might have little or no heritability and so cannot evolve. This is equivalent to the argument that the tumor microenvironment determines a tumor-propagating cell, which we continue to define as a cell capable of dividing symmetrically to generate two tumor-propagating daughter cells. The tumor microenvironment is obviously important (Bissell and Radisky 2001). Recent evidence in human lung cancer cells demonstrates that environmental cues, in the form of cell density, can affect the frequency of symmetric division (Pine et al. 2010), although it has also been observed that genetic mutations can affect the propensity for symmetric division in *Drosophila melanogaster* (Caussinus and Gonzalez 2005). Stemness likely has both genetic and environmental proximal causes, as the fitness effects of mutations depend on the environmental context. In support of this view, it has been shown in mouse models that the propensity for breast cancer cells to divide symmetrically and self-renew is affected by the mutational state of ErbB2 and p53 and that the relative ratio of symmetric-to-asymmetric tumor-propagating cell divisions may change over time in response to the tumor microenvironment (Cicalese et al. 2009). This hybrid determination of symmetric-to-asymmetric division suggests that even if the propensity to become a tumor-propagating cell is extrinsically modulated, cells will increase their fitness relative to others in their microenvironment via cell intrinsic mechanisms when possible. We tested whether tumor-propagating cells are still favored in a model in which the microenvironmental niche confers stemness and the propensity to self-renew has the opportunity to evolve independently (Supplementary Methods). To do so, we defined a location in the neoplasm as the niche, and any cell that occupied it became a tumor-propagating cell. Upon cell division, both daughter cells of a tumor-propagating cell located in a niche remained tumor-propagating cells. A tumor-propagating cell not in the niche always divided symmetrically; either both daughters remained tumor-propagating cells and thus self-renewed, or both daughters differentiated into transient cells. All cells had independent probabilities for self-renewal that could evolve, and the probability of self-renewal for tumor-propagating cells not located in the niche was initialized to 0. Using our model, we found that natural selection continues to favor tumor-propagating cells by evolving high levels of tumor-propagating cell self-renewal at cell division (Fig. 4), although it took about twice as long for tumor-propagating cells to dominate the tumor as under the standard model (Table S1). There was a significant difference between the evolved symmetric division levels for this niche model compared to the control case of no evolution (*t*-test, *P* = 10^−134^). Of course, if the proportion of transient cells that encounter the niche and become tumor-propagating cells is large, the selective pressure on self-renewal will drop. Like in the dedifferentiation model, most of the cells in the tumor would be tumor-propagating cells, including transient cells that have a high chance of encountering a niche and becoming a tumor-propagating cell. We can formally tease apart the contribution of genetics and environment to the propensity to divide symmetrically by measuring the broad-sense heritability of the trait, which is simply the contribution of genetic variability (as opposed to environmental variability) to the phenotypic variability using label retention or other techniques to distinguish between symmetric and asymmetric divisions, and measuring the variation in the proportion of symmetric divisions between clones. We expect to observe, as we showed in our model, that tumor-propagating cells are still favored under the environmental control of stemness.

Finally, perhaps transient cells are maintained because they contribute to the fitness of nearby (and genetically identical) tumor-propagating cells, or even to their proliferative units, analogous to the evolution of multicellularity. As with the transition to multicellularity, cells must associate nonrandomly and share their resources, and between-unit selection must be greater than within-unit selection, such that proliferative units behave as reproductive entities. The crypt structure forms an enclosure promoting the nonrandom association of its members. Furthermore, crypts may reproduce by fission (McDonald et al. 2006), and there is competition between crypts (Chao et al. 2008). This is an intriguing parallel, but the question of whether competition among proliferative units occurs in malignant neoplasms, and how those proliferative units might reproduce, is still open. To test whether positive feedback from clones to their tumor-propagating cell ancestors was sufficient to restrain the evolution of high tumor-propagating cell symmetric division probability and thus limit the percent of tumor-propagating cells, we extended our model to include multi-level selection. We implemented a survival advantage to the tumor-propagating cells from clonally related transient cells (Supplementary Methods), although with equal success, we could have implemented the fitness benefit through a reproductive advantage. Tumor-propagating cells with more clonally related transient cells die less frequently than those with fewer. We found that the fitness benefits provided from transient cells to their clonally related tumor-propagating cell were sufficient to maintain initial levels of tumor-propagating cell symmetric division (Fig. 4). The evolved symmetric division levels for this feedback model and the control case of no evolution were statistically indistinguishable (*t*-test, *P* = 0.2). These results show that multi-level selection within a neoplasm, implemented here as positive feedback to tumor-propagating cells from transient cells, is a viable solution to resolve the discordance between experimental observations and theoretical predictions.

## Discussion

In the conventional view, all cells in a neoplasm are capable of being tumor-propagating cells. In contrast, the cancer stem cell hypothesis posits that a fraction of cells in a neoplasm cannot act as tumor-propagating cells. The difference between these views is the presence or absence of transient cells with limited reproductive potential in the neoplasm. Here, we used an evolutionary modeling approach to address this controversy.

Previous models have focused on the effects of tissue design (Komarova 2005), differentiation (Nowak et al. 2003), stem cell numbers in leukemias (Dingli et al. 2007c), the cancer stem cell niche (Sottoriva et al. 2010), and (a)symmetric stem cell division (Dingli et al. 2007a) on the rate of evolution. On the other hand, our individual-based model focuses on the evolution of tumor-propagating cells and includes dedifferentiation, relatively quiescent stem cells, the role of the microenvironment, and multi-level selection. Also, to our knowledge, a stage-structured analytical approach has not been applied to neoplastic differentiating systems. Using a computational model that encodes the fundamental ideas of the cancer stem cell hypothesis—that only a subset of cells in a tumor can self-renew—we showed that somatic evolution favors increased numbers of tumor-propagating cells. This result was robust to the somatic mutation rate, number of transient cell divisions before terminal differentiation, size of the neoplasm, and presence of a stem cell niche/microenvironmental effects. Only under clonal feedback, low trait variability, and intermediate levels of dedifferentiation, did we find that the number of tumor-propagating cells in the population did not increase.

As we have shown, it is not the existence of stem cells that is difficult to explain, but the continued presence of the nonstem cells that are evolutionary dead ends unless one or more of the following explanations are true: the cancer stem cell hypothesis is wrong or current assays do not correctly capture the composition of a tumor, somatic evolution is too slow, a tumor-propagating cell has low heritability and thus is determined by its microenvironment, or the fitness of tumor-propagating cells is increased by clonally related transient cells. It is likely that each tumor type will vary as to the cause or the mechanism by which it retains transient cells. We suspect that we will find positive fitness feedback mechanisms from the transient cells to the tumor-propagating cells in AML either directly or via interactions with accessory or stromal cells in the bone marrow microenvironment, while it is possible that tumor-propagating cell detection methods will continue to improve in melanoma such that most cells may eventually be identified as tumor-propagating cells as has been suggested (Quintana et al. 2008).

Resolving this paradox has important clinical implications. Cancer stem cells are thought to be particularly difficult to eliminate clinically. The possibility that, in addition to cell-level selection, other evolutionary processes can affect the dynamics of cancer progression can have significant implications for cancer treatment.
