# Supplementary material for: An evolutionary explanation for the presence of cancer nonstem cells in neoplasms
Source: Evol Appl. 2012 Nov 26;6(1):92–101. doi: 10.1111/eva.12030 (PMC3567474; doi:10.1111/eva.12030)
Supplement: Supplementary file 1 [file eva0006-0092-SD1.pdf]

Kathleen Sprouffske, C. Athena Aktipis, Jerald P. Radich, Martin Carroll, Aurora M. Nedelcu and Carlo C. Maley. *An evolutionary explanation for the presence of cancer nonstem cells in neoplasms*

Supplementary Material

Table of Contents

Supplementary Figures 2
Supplementary Methods 6
Stage structured population model 6
Individual-based model design and implementation 8
Supplementary Tables 11
Supplementary Text 15
Supplementary References 16

Supplementary Figures

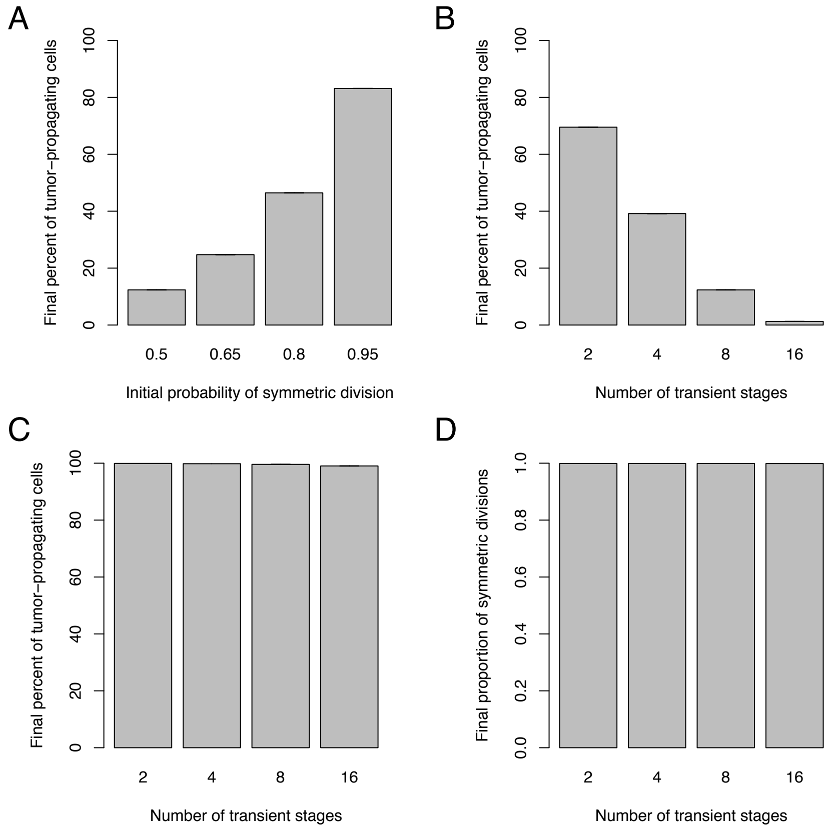

Fig. S1. The effect of varying the probability for symmetric division and the number of differentiation stages on the final percent of tumor-propagating cells and the proportion of tumor-propagating cell symmetric divisions.

(A) In the absence of evolution, increasing the probability for symmetric division is sufficient to increase the percent of tumor-propagating cells in a neoplasm. Simulations in which the probability of symmetric division could not evolve show that high levels of symmetric division result in high numbers of tumor-propagating cells. Values of less than 0.5 for the probability of symmetric division led to neoplasm extinction (n=[0,0,0,50,50,50,50] for probability of symmetric division=[0.05,0.2,0.35,0.5,0.65,0.8,0.95]). The probability of symmetric division and the final percent of tumor-propagating cells is highly correlated (Pearson's correlation R=0.97). (B) In the absence of evolution, increasing the number of differentiation stages decreases the percent of tumor-propagating cells in the neoplasm for populations with a fixed symmetric division probability of 0.5 (n=[49,50,49,26] for number transient cell stages=[2,4,8,16]). The number of transient stages and the final percent of tumor-propagating cells are negatively correlated (Pearson's correlation R=0.88). (C) In evolving populations, the number of differentiation stages did not affect the final percent of tumor-propagating cells or (D) the proportion of tumor-propagating cell symmetric divisions reached by natural selection (n=[50,50,47,29] for number transient cell stages=[2,4,8,16]). The proportion of tumor-propagating cell symmetric divisions was significantly higher than control simulations conducted in the absence of evolution (p=[$3x10^{-164}$,$2x10^{-167}$,$1x10^{-153}$,$3x10^{-79}$] for number transient cell stages=[2,4,8,16]). Error bars show standard error of the mean, 50 simulations were initialized per condition. Replicate simulations from each condition were similar and led to small error bars; in some cases the error bars are indistinguishable from the top of the data bar.

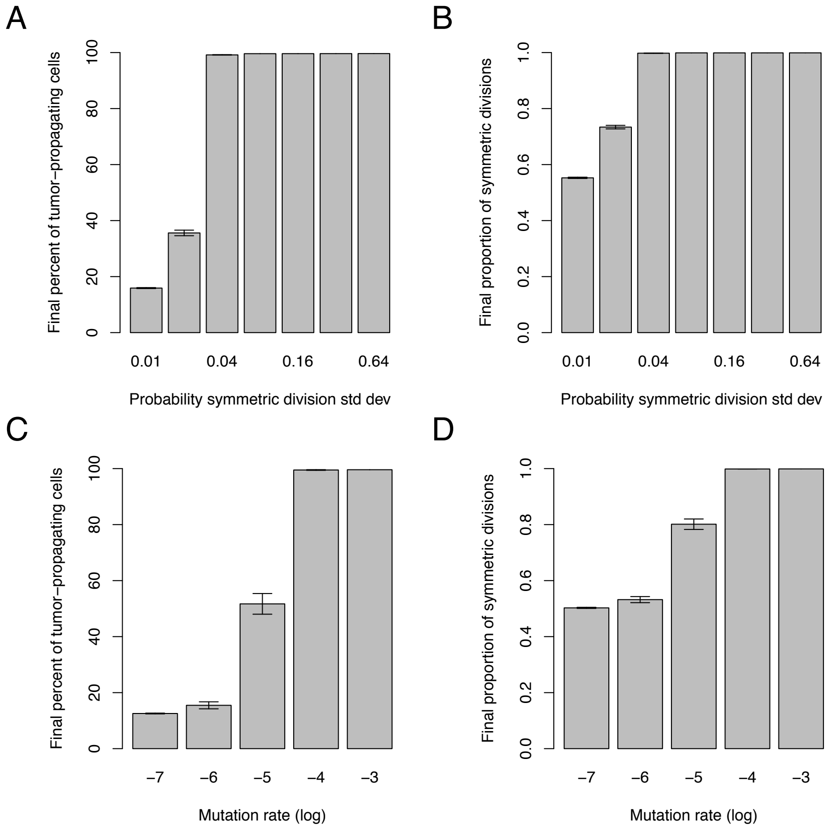

Fig. S2. Evolution of the tumor-propagating cell symmetric division trait requires sufficient variability for natural selection to act in 10 simulated years.

There was sufficient variability for the evolution of (A) high tumor-propagating cell and (B) symmetric division levels for the standard deviation of the symmetric division trait. The proportion of tumor-propagating cell symmetric divisions was significantly higher than control simulations conducted in the absence of evolution (p=[$5x10^{-28}$,$2x10^{-36}$,$6x10^{-120}$,$2x10^{-169}$,$6x10^{-166}$,$4x10^{-155}$,$3x10^{-169}$] for standard deviation=[0.01,0.02,0.04,0.08,0.16,0.32,0.64]). While the proportion of symmetric divisions did evolve under conditions of low trait variability, its rate of evolution was slow enough that there was insufficient time for the population to reach the high levels observed once it exceeded 0.04 (n=[48,47,47,48,47,48,48] for standard deviation=[0.01,0.02,0.04,0.08,0.16,0.32,0.64]). There was insufficient variability for the evolution of (C) high tumor-propagating cell and (D) symmetric division levels at low mutation rates as compared to control simulations conducted in the absence of evolution using a t-test with significance levels adjusted using the Bonferroni correction for multiple-testing (p=[0.08,0.002], n=[48,48] for mutation rate=[$1x10^{-7}$,$1x10^{-6}$]). . However, the mutation rate did not affect the final percent of tumor-propagating cells or the proportion of tumor-propagating cell symmetric divisions reached by natural selection once it exceeded $1x10^{-4}$ mutations in the asymmetric / symmetric division gene set per cell division (p=[$4x10^{-21}$,$1x10^{-112}$,$3x10^{-161}$] n=[48,48,50] for mutation rate=[$1x10^{-5}$,$1x10^{-4}$,$1x10^{-3}$]). Together, the mutation rate and the standard deviation of new mutations in the probability of symmetric division can define whether the symmetric division trait can evolve quickly enough for tumor-propagating cells to take over the neoplasm. Due to computational limitations, we did not run the simulation at realistic population sizes (10^9-10^{12}), however, with larger populations, lower mutation rates result in the evolution of high symmetric division probabilities and tumor-propagating cells will continue to dominate the neoplasm. Error bars show standard error of the mean, 50 simulations were initialized per condition.

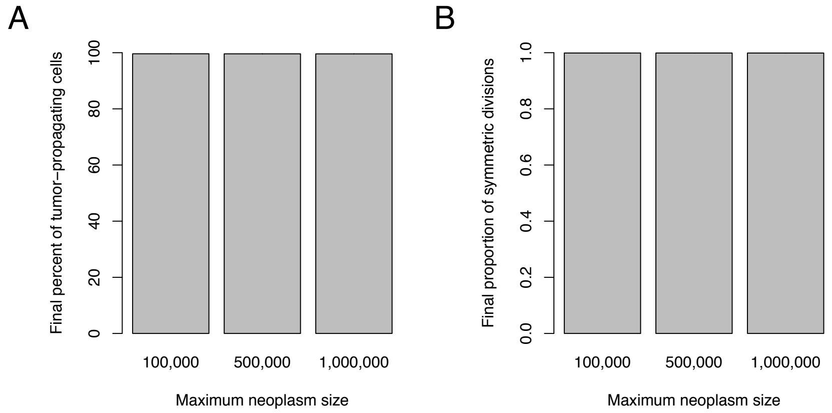

Fig. S3. All maximum possible neoplasm sizes, or tissue carrying capacities, resulted in the evolution of (A) high tumor-propagating cell levels and (B) high tumor-propagating cell symmetric division probabilities, and was significantly higher than control simulations conducted in the absence of evolution (p=[$4x10^{-155}$,$3x10^{-161}$,$3x10^{-161}$] and n=[48,50,50] for maximum neoplasm size=[$1x10^{5}$,$5x10^{5}$,$1x10^{6}$]). Error bars show standard error of the mean, 50 simulations were initialized per condition.

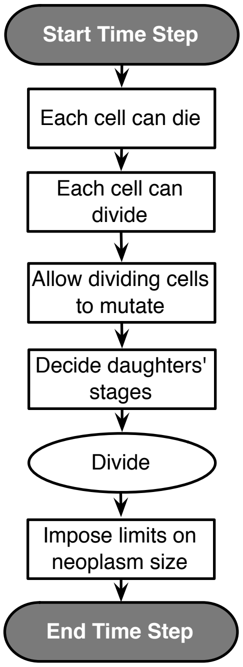

Fig. S4. Flow chart depicting the order of events that occurs each day during a simulation. Each box is described in detail in the Supplementary Methods Submodels section.

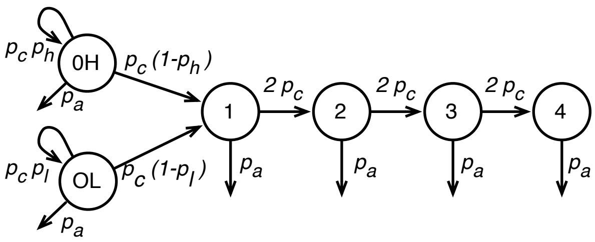

Fig. S5. A graphical representation of the transitions between stages associated with the analytical stage-structured population model. Tumor-propagating cells with high symmetric division rates, p_h, are labeled 0H and those with lower symmetric division rates, p_l, are labeled 0L. Transient cells, labelled 1-3, differentiate at cell division until they terminally differentiate and reach stage 4. The apoptosis rate p_a and the cell division rate p_c are constant across all stages. Note that one cell in transient stage *s-1* divides to become 2 cells in stage *s*, which is not depicted graphically but is included in the population projection matrix **A** in the Materials and Methods.

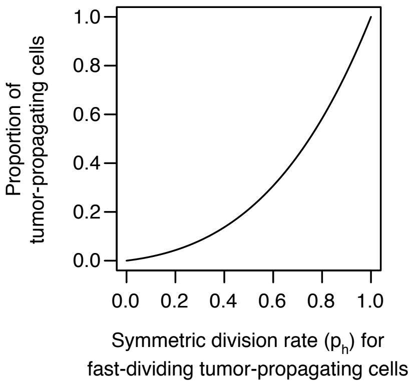

Fig. S6. Increasing the symmetric division rate for the fast-dividing tumor-propagating cells causes an increase in the proportion of tumor-propagating cells. The proportion of tumor-propagating cells in the neoplasm is given by $\frac{1}{8}p_h(1+p_h)^3$ and is plotted here.

Supplementary Methods

Stage structured population model

In our analytical, deterministic approach, we constructed a stage-structured population model (Lefkovitch 1965; Caswell 2001) to characterize the stable stage distribution of differentiation states. To do so, we split the tumor-propagating cells into two groups: stages 0H and 0L and assigned stage 0H a higher symmetric division rate p_h than stage 0L p_l, such that $p_h > p_l$. We then observed the outcome on the asymptotic population composition of “competition” between clones of tumor-propagating cells with different symmetric division rates. Transient and differentiated cells were defined as depicted in Fig. S5.

We constructed a stage-classified matrix model, in which $\mathbf{n}(t+1) = \mathbf{A}\,\mathbf{n}(t)$, where $\mathbf{n}(t)$ is a vector of stage abundances at time t and $\mathbf{A}$ is the population projection matrix. We depict our model both graphically (Fig. S5) and mathematically using the population projection matrix $\mathbf{A}$,

$$\mathbf{A} = \begin{pmatrix} 1-p_a-p_cp_h & 0 & 0 & 0 & 0 & 0 \\ 0 & 1-p_a-p_cp_l & 0 & 0 & 0 & 0 \\ p_c(1-p_h) & p_c(1-p_l) & 1-p_c-p_a & 0 & 0 & 0 \\ 0 & 0 & 2p_c & 1-p_c-p_a & 0 & 0 \\ 0 & 0 & 0 & 2p_c & 1-p_c-p_a & 0 \\ 0 & 0 & 0 & 0 & 2p_c & 1-p_a \end{pmatrix}.$$

This accounts for both the rate of transition between stages and the number of individuals affected. A matrix entry a_{ij} can be interpreted as the contribution of cell abundances from stage j at time t to the cell abundances in stage i at time $t + 1$. For example, the number of cells in stage 1 at time $t+1$ is given by, $n_1(t+1) = n_{oH}(t)\, p_c\,(1-p_h) + n_{oL}(t)\, p_c\,(1-p_l) + n_1(t)\,(1 - p_c - p_a)$. The rest of the parameters in $\mathbf{A}$ are as previously defined (Table S2), where p_a is the rate of apoptosis per day and p_c is the rate of cell division per day.

The dominant eigenvalue λ_1 of matrix $\mathbf{A}$ corresponds to the change in population size over time and is constant when $\lambda_1 = 1$. When $\lambda_1 > 1$, the tumor size increases and when $\lambda_1 < 1$, the tumor shrinks. The dominant eigenvalue λ_1 of the characteristic equation of $\mathbf{A}$ is $\lambda_1 = 1 - p_a - p_cp_h$, since $p_h > p_l$. Thus, tumor growth depends on the apoptosis and cell division rates and only the higher symmetric division rate. Using our default parameter values for apoptosis and cell division (Table S2), the tumor size is constant when $p_h = 0.41176$, grows exponentially when $p_h > 0.41176$, and shrinks to extinction when $p_h < 0.41176$. This is consistent with the observation in our agent-based model that small initial probabilities of symmetric divisions could not sustain a tumor population (Fig. S1).

The right eigenvector of λ_1 gives the asymptotic distribution of cells in the stage classes and in the general case is

$$\mathbf{w_1} = \begin{pmatrix} \frac{p_h(1+p_h)^3}{8(1-p_h)} & 0 & \frac{1}{8}p_h(1+p_h)^2 & \frac{1}{4}p_h(1+p_h) & \frac{1}{2}p_h & 1 \end{pmatrix}^T.$$

From this, we observe that the asymptotic population distribution value for stage 0L is 0 for all values of p_a, p_c, p_h, and p_l, and thus cells with the lower symmetric division rates do not survive. This is consistent with the evolutionary simulations from our individual-based model, in which mutant clones with lower symmetric division rates eventually reached extinction. The actual dynamics of this cell population depend on the initial conditions, in that there must be cells in stage 0H.

We can calculate the proportion of the neoplasm that comprises tumor-propagating cells from the aymptotic population distribution by dividing the fraction of the cells that are in stage 0H by the sum of the

fraction of cells in any stage. Doing this, we find that the fraction of tumor-propagating cells in the neoplasm depends only on the symmetric division rate for fast-dividing tumor-propagating cells and is given by

$$\frac{1}{8}p_h(1+p_h)^3.$$

Thus, the proportion of tumor-propagating cells in the neoplasm increases as the tumor-propagating cell's rate of symmetric division, p_h, increases (Fig. S6).

Individual-based model design and implementation

These methods are presented in ODD protocol standard format for individual-based models (Grimm et al. 2006). Briefly, tumor-propagating cells and transient cells were modeled using NetLogo 4.0.4 (Wilensky 1999). Each day in the simulation, a cell had the opportunity to die, divide, or do nothing. Transient cells could divide symmetrically a parameterized number of times before undergoing apoptosis or senescence. Except where noted, transient cells could not dedifferentiate into tumor-propagating cells. The probability that a tumor-propagating cell divided symmetrically or asymmetrically was determined by a mutable cell intrinsic trait that could evolve.

Model overview

Purpose

This individual-based model tests how the probability for symmetric division evolves in tumor-propagating cells, or cancer stem cells, given the cancer stem cell hypothesis of cancer.

Entities, state variables, and scales

Each cell in a simulation has values for the state variables defined in Table S2.

Process overview and scheduling

The model is broken into timesteps, each of which is equivalent to 1 day. The flow chart in Fig. S4 outlines the decisions that a cell makes each timestep. Details of the implementation are described in the Submodels below.

Adaptation

Cells in this model are not explicitly encoded with adaptive behavior. Instead, increasing propensity of symmetric division implicitly increases the fitness of the cell by decreasing the rate at which cells become fully differentiated and are removed from the population.

Sensing

Cells are not encoded with any sensing ability.

Interaction

There are no direct interactions or communication between cells in the model.

Stochasticity

Cell division, cell death, and mutation are stochastic in the model. They are constrained by parameterized probabilities.

Observation

For all simulations, summary data are recorded at the end of the simulation. For certain simulations, summary data are written to a file every 10 days. These data include the number of tumor-propagating cells, the number of transient cells, the observed average propensity for symmetric division by tumor-propagating cells, and the time at which simulation crossed the 90% tumor-propagating cells threshold.

Model details

Initialization

See Table S2 for the parameter values and their ranges used in the simulations. Simulations are initialized with n_s tumor-propagating cells and n_t transient cells. A cell i is a tumor-propagating cell at time t if its stage is 0, $s_i(t) = 0$. A cell i is a transient cell at time t if its stage is not 0, $s_i(t) > 0$. All cells have the same

propensity for cell division p_c and apoptosis p_a. Tumor-propagating cells can divide symmetrically or asymmetrically. Both daughter cells from a symmetric division remain tumor-propagating cells. When a tumor-propagating cell divides asymmetrically, one daughter cell remains a tumor-propagating cell and the other differentiates into a transient cell (Fig. 1A-C). Initially, each cell *i* has probability $p_{sd,i}(0) = p_{sd}$ of symmetric division if it is a tumor-propagating cell. Each cell's probability of symmetric division $p_{sd,i}(t)$ is allowed to evolve over time. Because a tumor-propagating cell can only divide symmetrically or asymmetrically, allowing the probabilities of symmetric division or asymmetric division to evolve is equivalent.

Submodels

Each submodel corresponds to a decision in the flowchart (Fig. S4).

Each cell can die

For most of the simulations, a cell dies from background mortality if it draws a random number from a uniform distribution between 0 and 1 that is less than the probability for apoptosis, p_a.

In a subset of the simulations that test the effects of feedback from transient cells to stem cells, transient cells die as before while stem cells with higher numbers of clonal transient cells die less often than those with fewer. The number of clonal transient cells for a stem cell *i* at time *t*, or $n_{c,i}(t)$, is defined as the number of transient cells that arise from the most recent common stem cell ancestor (as such, the stem cell and its clonal transient cells have common mutational state). Specifically, a stem cells dies if it draws a random number from a uniform distribution between 0 and 1 that is less than $p_{af}(t)$, the probability for apoptosis under feedback at time *t*, where $p_{af}(t)$ is obtained from the sigmoid function

$$p_{af}(t) = \frac{1}{1 + \left(\frac{1}{p_a}\right) e^{-rn_{c,i}(t)}},$$

and *r* is an arbitrary constant (*r* = -0.0001). Varying negative values of *r* had no effect on the outcome of the simulations.

A cell that is fully differentiated dies. The number of differentiation stages is a parameter *s* to the model, and each cell *i* has a state variable that tracks its differentiation level at time *t*, $s_i(t)$. Thus, cells with $s_i(t) = s$ die. The qualitative results of the model do not change if terminally differentiated cells are allowed to remain as quiescent cells in the model and are slowly cleared by background apoptosis.

Each cell can divide

A cell divides if it draws a random number from the uniform distribution between 0 and 1 that is less than the probability for cell division, p_c.

Allow dividing cells to mutate

A cell *i* acquires a mutation in its probability for its tumor-propagating symmetric cell division trait if it draws a number from a uniform distribution between 0 and 1 that is less than the mutation rate, μ. Once it gets a new mutation, the current probability for symmetric division for its daughters, cells *j* and *k*, are updated to $p_{sd,j}(t+1) = p_{sd,k}(t+1) = N(p_{sd,i}(t),\sigma)$, where the mean of the normal distribution is the parental probability of symmetric division, $p_{sd,i}(t)$, and σ is the standard deviation parameter. The updated probabilities for symmetric division are bounded by 0 and 1.

Decide daughters' differentiation level

Recall that the differentiation stage for a cell *i* at time *t* is stored in its local variable $s_i(t)$. Here, we determine the differentiation stage for daughter cells *j* and *k*. In most simulations, a tumor-propagating cell can divide symmetrically or asymmetrically. It divides symmetrically if it draws a random number from a uniform distribution between 0 and 1 that is less that its current probability for symmetric division $p_{sd,i}(t)$, and asymmetrically otherwise. When a tumor-propagating cell divides symmetrically, both of its daughters remain tumor-propagating cells, $s_j(t+1) = s_k(t+1) = 0$. When a tumor-propagating cell divides

asymmetrically, one daughter remains a tumor-propagating cell, $s_j(t+1) = 0$, and the other differentiates into a transient cell, $s_k(t+1) = 1$.

A transient cell divides symmetrically. Most of the simulations were run under a normal, or forward-only differentiation regime, in which the progeny from transient cells were always more differentiated, $s_j(t+1) = s_k(t+1) = s_i(t) +1$. In a subset of the simulations, transient cells were allowed to dedifferentiate according to a parameterized probability, such that both daughters became tumor-propagating cells, $s_j(t+1) = s_k(t+1) = 0$.

Impose limits on neoplasm size

If the total number of cells in the neoplasm N exceeds the maximum possible carrying capacity of the tissue n_{max}, then $N - n_{max}$ cells are chosen at random for death until the total population is n_{max}. The total number of cells in a simulation fluctuates over the course of each timestep, typically decreasing when the cell death submodels are executed and increasing when the cell birth submodels is executed.

Niche-determination of tumor-propagating cells

We extended the basic model to explore the effects of microenvironmentally-determined "stemness", in which the differentiation structure remains, and any cell that moves into the niche becomes a tumor-propagating cell. Tumor-propagating cell self-renewal is defined as a symmetric division in which both daughter cells remain tumor-propagating cells, and has a probability of occurring in cell i at time t of $p_{sr,i}(t)$. Here, we implement the mutation and evolution of self-renewal just as we implemented mutation of symmetric division in the basic model. All cells are placed on the neoplasm at (0,0) in a 32x32 torus, and the niche is located at (0,0). At cell division, one daughter remains in place and the other moves 1 niche-width in a random direction. Any transient cell i that moves into the niche becomes a tumor-propagating cell, such that $s_i(t+1) = 0$. Both daughters for a tumor-propagating cell in the niche remain tumor-propagating cells. Tumor-propagating cells not in the niche can only divide symmetrically, and so both daughters j and k are either tumor-propagating cells $s_j(t+1) = s_k(t+1) = 0$ or transient cells $s_j(t+1) = s_k(t+1) = 1$. A tumor-propagating cell not in the niche self-renews if it draws a random number from a uniform distribution between 0 and 1 that is less that its current probability for self-renewing division $p_{sr,i}(t)$, and differentiates otherwise. The initial probability for tumor-propagating cell self-renewing division is zero for all cells. All other transient cells differentiate as previously defined.

Supplementary Tables

Table S1. Summary data for all of the experimental conditions simulated.

Mean final probability for symmetric division and percent of tumor-propagating cells. Fifty simulations were initiated for each experimental condition, including the default parameter values (see Table S2), control experiments in which the default parameters were used but mutation was not allowed, and varying each of the parameter values. The percent of tumor-propagating cells, the population's mean probability for symmetric division, and the time to 90% tumor propagating cells were recorded. The mean and standard error are reported for these metrics. Most parameter values resulted in symmetric division rates that were statistically greater than the control simulation cases (marked *). Significance was obtained using a t-test with the Bonferroni multiple-testing correction (significant at $p < 0.0016$). Due to the nature of stochastic simulation modeling, sometimes populations went extinct before the simulation completed. The number of simulations per experiment that survived for 10 years is reported.

Experiment number	Parameter value	Probability for symmetric division		P-value (* denotes significance)	Percent of tumor-propagating cells		Time to 90% tumor propagating cells (years)		Number of simulations	
		Mean	Standard error		Mean	Standard error	Mean	Standard error	Reached 90%	Completed
Default parameters (see Table S2)										
	n_s=50<br>n_t=99,950<br>p_c=0.17<br>p_a=0.07<br>μ=1x10^{-3}<br>p_{sd}=0.5<br>σ=0.08<br>p_d=0<br>d=8<br>n_{max}=100,000	0.99892	0.00003	3 x 10^{-164} *	99.6	0.01	2.97	0.07	49	49
Control using default parameters (see Table S2)										
2	μ=0	0.5	0	control	12.42	0.03	N/A	0 / 49	0	49

Experiment number	Parameter value	Probability for symmetric division		P-value (* denotes significance)	Percent of tumor-propagating cells		Time to 90% tumor propagating cells (years)		Number of simulations	
		Mean	Standard error		Mean	Standard error	Mean	Standard error	Reached 90%	Completed
Probability of dedifferentiation										
3	$p_d=0$	0.99888	0.00004	4×10^{-155} *	99.6	0.02	2.97	0.07	48	48
4	$p_d=0.2$	0.97323	0.0025	4×10^{-72} *	94.88	0.45	8.36	0.13	47	50
5	$p_d=0.4$	0.52914	0.00113	2×10^{-30} *	63.32	0.06	N/A	N/A	0	50
6	$p_d=0.6$	0.50636	0.00095	1×10^{-08} *	70.86	0.05	N/A	N/A	0	50
7	$p_d=0.8$	0.50309	0.00092	8×10^{-04} *	76.31	0.04	N/A	N/A	0	50
8	$p_d=1$	0.50302	0.00082	3×10^{-04} *	80.09	0.03	N/A	N/A	0	50
Relative quiescence of tumor-propagating cells										
9	$p_{c\text{-}tpc} / p_{c\text{-}trans} = 0.425$	0.9989	0.00003	8×10^{-126} *	99.04	0.03	3.75	0.11	36	37
10	$p_{c\text{-}tpc} / p_{c\text{-}trans} = 0.567$	0.99895	0.00003	2×10^{-151} *	99.29	0.02	3.66	0.09	45	45
11	$p_{c\text{-}tpc} / p_{c\text{-}trans} = 1$	0.99894	0.00002	5×10^{-176} *	99.62	0.01	2.8	0.07	50	50
Number of stages										
12	$d=2$	0.99893	0.00003	3×10^{-164} *	99.93	0.002	2.17	0.04	49	49
13	$d=4$	0.99892	0.00003	2×10^{-167} *	99.82	0.005	2.51	0.06	50	50
14	$d=8$	0.99889	0.00005	1×10^{-153} *	99.59	0.02	3.02	0.07	49	49
15	$d=16$	0.99875	0.00008	3×10^{-79} *	99.03	0.06	4.31	0.17	26	26

Experiment number	Parameter value	Probability for symmetric division		P-value (* denotes significance)	Percent of tumor-propagating cells		Time to 90% tumor propagating cells (years)		Number of simulations	
		Mean	Standard error		Mean	Standard error	Mean	Standard error	Reached 90%	Completed
Standard deviation of probability symmetric division										
16	$\sigma=0.01$	0.5527	0.00223	5 x 10^{-28} *	15.9	0.17	N/A	N/A	0	48
17	$\sigma=0.02$	0.73384	0.00627	2 x 10^{-36} *	35.59	0.99	N/A	N/A	0	47
18	$\sigma=0.04$	0.99777	0.0002	6 x 10^{-120} *	99.17	0.08	6.58	0.11	47	47
19	$\sigma=0.08$	0.99895	0.00002	2 x 10-^{169} *	99.61	0.01	2.95	0.06	48	48
20	$\sigma=0.16$	0.99894	0.00002	6 x 10^{-166} *	99.62	0.01	1.8	0.06	47	47
21	$\sigma=0.32$	0.99895	0.00004	4 x 10^{-155} *	99.62	0.01	1.18	0.06	48	48
22	$\sigma=0.64$	0.99899	0.00002	2 x 10^{-169} *	99.64	0.01	0.74	0.05	48	48
Mutation rate										
23	$\mu=1x10^{-7}$	0.50272	0.00193	8 x 10^{-02}	12.55	0.14	N/A	N/A	0	48
24	$\mu=1x10^{-6}$	0.53212	0.01084	2 x 10^{-03}	15.47	1.25	N/A	N/A	0	48
25	$\mu=1x10^{-5}$	0.80144	0.01867	4 x 10^{-21} *	51.7	3.69	8.42	0.43	7	48
26	$\mu=1x10^{-4}$	0.99865	0.00032	1 x 10^{-112} *	99.5	0.12	5.45	0.18	48	48
27	$\mu=0.001$	0.99892	0.00004	3 x 10^{-161} *	99.6	0.02	2.99	0.08	50	50
Maximum neoplasm size										
28	$n_{max}=100,000$	0.99894	0.00004	4 x 10^{-155} *	99.62	0.02	3.06	0.06	48	48
29	$n_{max}=500,000$	0.99891	0.00004	3 x 10^{-161} *	99.6	0.01	2.49	0.04	50	50
30	$n_{max}=1,000,000$	0.99887	0.00004	3 x 10^{-161} *	99.59	0.02	2.3	0.03	50	50
Microenvironmental modulation of tumor-propagating cells (niche model)										
31		0.99816	0.00014	1 x 10^{-134} *	98.66	0.1	6.9	0.16	50	50
Fitness increase to tumor-propagating cells from clonally-related transient cells										
32		0.502	0.002	2 x 10^{-01}	33	0.2	N/A	N/A	0	50

Table S2. Parameter values for our model of tumor-propagating cell symmetric division.

Parameter	Description	Biological value	Model value (default value)
Number tumor-propagating cells (n_s)	Initial number of tumor-propagating cells	13,250 - 353,000 cells (Rubinow and Lebowitz 1976)	50 - 500 cells (50)
Number transient cells (n_t)	Initial number of transient cells	≈ $5x10^{10}$ cells (Rubinow and Lebowitz 1976)	99,950 - 999,500 cells (99,950)
Probability of cell division (p_c)	The probability a given cell divides each day	0.173 divisions / day (Rubinow and Lebowitz 1976)	0.17 cell divisions / day (0.17)
Probability of apoptosis (p_a)	The probability a given cell dies each day	0.071 deaths / day (Rubinow and Lebowitz 1976; Mundle et al. 1994)	0.07 deaths / day (0.07)
Mutation rate (μ)	The chance of a cell acquiring a mutation each cell division	$1.6x10^{-8}$ - $1x10^{-3}$ mutations / cell division (Araten et al. 2005; Bielas and Loeb 2005; Siegmund et al. 2009; Ushijima et al. 2005)	$1x10^{-7}$- $1x10^{-3}$ mutations / cell division ($1x10^{-3}$)
Probability of symmetric division (p_{sd})	Initial probability that a given tumor-propagating cell's daughters are both tumor-propagating cells, each cell division	Unknown	0.05 - 0.95 symmetric / cell division (0.5)
Standard deviation (σ)	Standard deviation of probability of tumor-propagating cell symmetric division	Unknown	0.01 - 0.64 (0.08)
Probability of dedifferentiation (p_d)	Probability that a given transient cell's daughters are both tumor-propagating cells, each cell division	Unknown	0 - 1 / cell division (0)
Number of stages (d)	Number of possible differentiation stages	Minimum 8 stages, if AML mimics hematopoiesis (Khanna-Gupta and Berliner 2005; Ansker et al. 2005)	2 - 16 stages (8)
Maximum neoplasm size (n_{max})	Maximum size possible for a neoplasm	Model-specific	100,000 - 1,000,000 cells (100,000)

Table S3. State variables for our model of tumor-propagating cell symmetric division.

Parameter	Description
Stage ($s_i(t)$)	Differentiation stage of cell *i* at time *t*, characterizes if cell *i* is a tumor-propagating cell or any stage of transient cell
Probability of tumor-propagating cell division ($p_{sd,i}(t)$)	The probability that a tumor-propagating cell *i* divides symmetrically at time *t*, once it divides

Supplementary Text

Natural selection continues to favor a high tumor-propagating cell symmetric division probability, regardless of the number of differentiation stages.

In normal hematopoiesis, there are at least 8 distinct maturation stages, or levels of differentiation, in the neutrophil lineage (Khanna-Gupta and Berliner 2005). It is not known if the levels of differentiation in AML mimic that of hematopoiesis, or how many times a transient cell in AML may divide before it terminally differentiates. Thus, we varied the number of times a cell could divide before terminal differentiation. In experiments with constant probability for tumor-propagating symmetric divisions (i.e., no evolution), we found that increasing the number of differentiation stages decreases the percent of tumor-propagating cells in the neoplasm (Fig. S1B). Showing the robustness of our model by varying the number of differentiation stages, we found that natural selection still favored high chances of tumor-propagating cell symmetric divisions (Fig. S1D), and thus high levels of tumor-propagating cells (Fig. S1C).

Natural selection continues to favor a high tumor-propagating cell symmetric division probability as long as there is sufficient variability in the tumor-propagating cell symmetric division trait.

Mutation in the probability of symmetric division was modeled at cell division as a two-step process. Each cell could either mutate or not, and if it did then its current probability for symmetric division was updated by drawing a random number from a normal distribution centered on the parental cell's symmetric division rate with a parameterized standard deviation (see Supplementary Methods). Because the standard deviation of the tumor-propagating cell symmetric division rate is unknown, we tested a range of values. We also varied the mutation rate across physiologically reasonable ranges. We found that when the mutation rate or the effects of mutations are extremely small, the tumor-propagating cell's symmetric division probability trait does not evolve during the 10-year experiment. However, with sufficient variability in the symmetric division trait, natural selection continues to favor a high tumor-propagating cell symmetric division probability and high levels of tumor-propagating cells (Fig. S2).

Natural selection continues to favor a high tumor-propagating cell symmetric division probability, regardless of the maximum neoplasm size.

To keep the model computationally tractable, default *in silico* neoplasms had resources to maintain 100,000 cells. When a neoplasm reached this carrying capacity, cells were randomly selected to undergo apoptosis until the carrying capacity was again met. We varied the carrying capacity of the neoplasm and found that the population size of the neoplasm has no effect on either the evolution of the probability of symmetric division, or the final proportion of tumor-propagating cells in the neoplasm (Fig. S3).

Supplementary References

Ansker, Y., H. Leung, and C. Vergaillie. 2005. Tumor-propagating cell model of hematopoeisis. In *Hematology: basic principles and practices*, edited by R. E. A. Hoffman. Philadelphia: Elsevier.

Araten, D. J., D. W. Golde, R. H. Zhang, H. T. Thaler, L. Gargiulo, R. Notaro, and L. Luzzatto. 2005. A quantitative measurement of the human somatic mutation rate. Cancer Research **65**:8111-7.

Bielas, J. H., and L. A. Loeb. 2005. Quantification of random genomic mutations. Nat Methods **2**:285-90.

Caswell, H. 20001. *Matrix population models*. 2nd ed: Sinauer Associates, Sunderland, MA.

Grimm, V., U. Berger, F. Bastiansen, S. Eliassen, V. Ginot, J. Giske, J. Goss-Custard et al. 2006. A standard protocol for describing individual-based and agent-based models. Ecological Modelling **198**:115-126.

Khanna-Gupta, A., and N. Berliner. 2005. Granulocytopoiesis and monocytopoeisis. In *Hematology: Basic Principles and Practice*, edited by R. E. A. Hoffman. Philadelphia: Elsevier.

Lefkovitch, L. 1965. The study of population growth in organisms grouped by stages. Biometrics 21:1-18.

Mundle, S., A. Iftikhar, V. Shetty, S. Dameron, V. Wright-Quinones, B. Marcus, J. Loew et al. 1994. Novel in situ double labeling for simultaneous detection of proliferation and apoptosis. Journal of Histochemistry and Cytochemistry **42**:1533-7.

Rubinow, S. I., and J. L. Lebowitz. 1976. A mathematical model of the acute myeloblastic leukemic state in man. Biophysical Journal **16**:897-910.

Siegmund, K. D., P. Marjoram, Y.-J. Woo, S. Tavaré, and D. Shibata. 2009. Inferring clonal expansion and cancer stem cell dynamics from DNA methylation patterns in colorectal cancers. Proceedings of the National Academy of Sciences of the United States of America **106**:4828-33.

Ushijima, T., N. Watanabe, K. Shimizu, K. Miyamoto, T. Sugimura, and A. Kaneda. 2005. Decreased fidelity in replicating CpG methylation patterns in cancer cells. Cancer Research **65**:11-7.

Wilensky, U. 1999. NetLogo. http://ccl.northwestern.edu/netlogo/ Center for Connected Learning and Computer-Based Modeling, Northwestern University, Evanston, IL.
