# Supplementary figures and images for: An evolutionary explanation for the presence of cancer nonstem cells in neoplasms
Source: Evol Appl. 2012 Nov 26;6(1):92–101. doi: 10.1111/eva.12030 (PMC3567474; doi:10.1111/eva.12030)

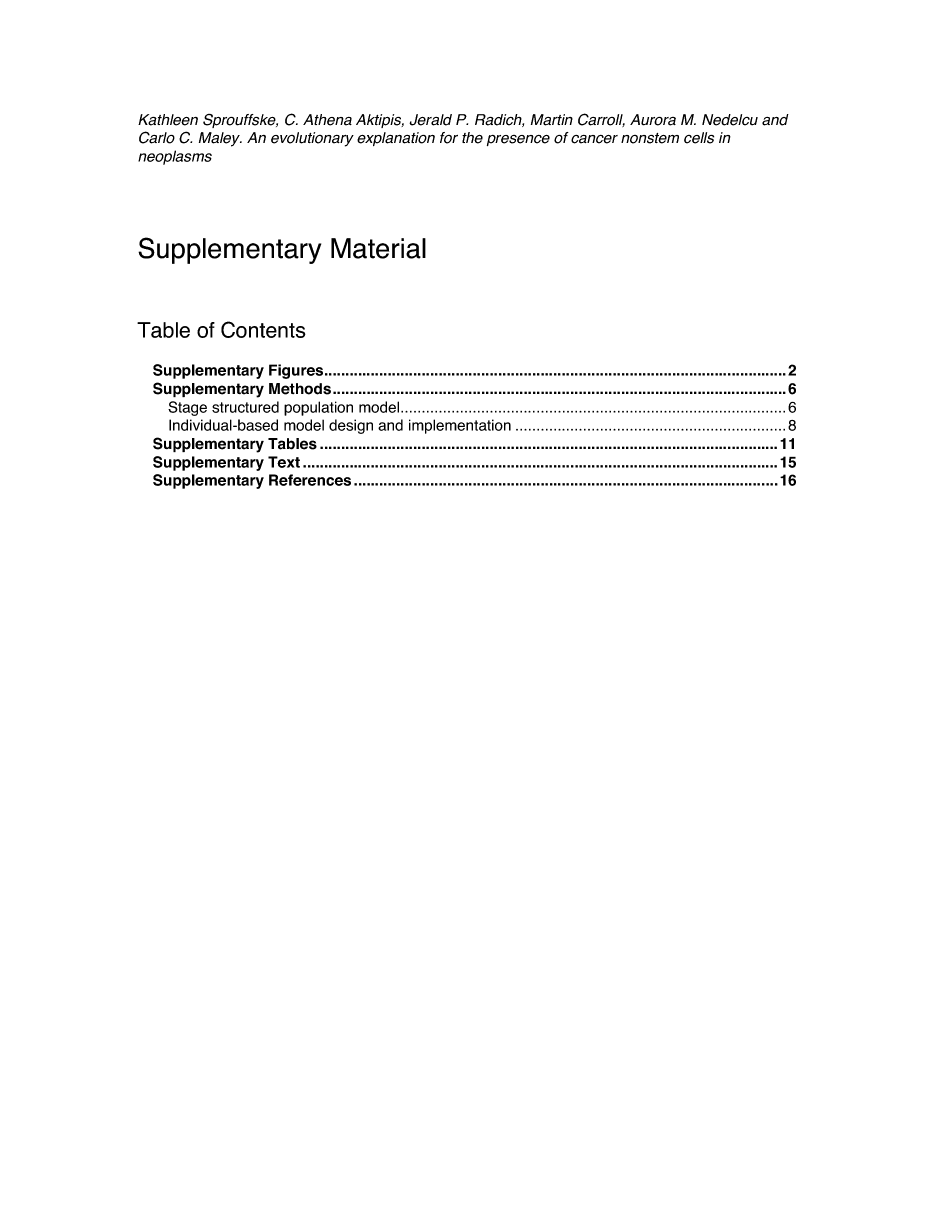

Supplement: Supplementary file 2 [file eva0006-0092-SD2.png]
